# Short progression‐free survival of ALK inhibitors sensitive to secondary mutations in ALK‐positive NSCLC patients

**DOI:** 10.1111/1759-7714.13143

**Published:** 2019-07-23

**Authors:** Naoki Haratake, Takashi Seto, Shinkichi Takamori, Ryo Toyozawa, Kaname Nosaki, Naoko Miura, Taro Ohba, Gouji Toyokawa, Kenichi Taguchi, Masafumi Yamaguchi, Mototsugu Shimokawa, Mitsuhiro Takenoyama

**Affiliations:** ^1^ Department of Thoracic Oncology National Hospital Organization Kyushu Cancer Center Fukuoka Japan; ^2^ Department of Thoracic Surgery National Hospital Organization Kyushu Medical Center Fukuoka Japan; ^3^ Department of Pathology National Hospital Organization Kyushu Cancer Center Fukuoka Japan; ^4^ Department of Biostatistics Yamaguchi University Graduate School of Medicine Yamaguchi Japan; ^5^ Clinical Research Institute National Hospital Organization Kyushu Cancer Center Fukuoka Japan

**Keywords:** ALK, non‐small cell lung Cancer, re‐biopsy, resistance, sequential treatment

## Abstract

**Background:**

Most non‐small cell lung cancer (NSCLC) patients relapse on anaplastic lymphoma kinase‐tyrosine kinase inhibitor (ALK‐TKI) therapy because of acquired resistance. Rebiopsy is recommended to provide optimal therapy after relapse for some ALK‐TKI therapies; however, little clinical data exists on the clinical efficacy of ALK‐TKI tailored to secondary mutation.

**Methods:**

A retrospective study was conducted to analyze the patterns of ALK‐TKI treatment and clinical outcomes, including progression free survival (PFS), of ALK‐positive NSCLC patients who received rebiopsy. Based on the rebiopsy results, secondary mutations in the ALK gene that were shown to be associated with the efficacy of ALK‐TKI therapy in the preclinical or clinical setting were defined as “sensitive mutations (SM)”.

**Results:**

Among 71 patients who received ALK‐TKI for NSCLC at our institution, 20 patients received rebiopsy, and secondary SM were found in eight patients. The objective response rate (ORR) of the cases with SM who received ALK‐TKI therapy was 88.9%, while the ORR of the patients without SM who received ALK TKI or chemotherapy was 20.0%; however, the PFS of the patients with SM was relatively short (with SM vs. without SM: 5.6 months vs. 5.1 months).

**Conclusions:**

The selection of ALK‐TKI based on the rebiopsy result was associated with a high ORR and relatively short PFS. The mechanism responsible for the short PFS of sensitive ALK‐TKI to secondary mutation should be clarified.

## Introduction

Lung cancer is the leading cause of cancer‐related death worldwide. Recent advances in chemotherapy and molecular targeted therapy have led to the remarkable improvement of survival of lung cancer patients, especially those with non‐small cell lung cancer (NSCLC). From 3%–5% of NSCLC patients have anaplastic lymphoma kinase (ALK) rearrangement. The superiority of crizotinib, a first generation ALK‐tyrosine kinase inhibitor (ALK‐TKI), to standard chemotherapy has been demonstrated in patients with ALK‐positive advanced NSCLC previously treated with platinum‐based chemotherapy.^1^ Sequentially, next generation ALK‐TKIs, such as alectinib, ceritinib, brigatinib and lorlatinib, have shown remarkable clinical efficacy in ALK‐positive NSCLC.^2^, ^3^, ^4^, ^5^, ^6^, ^7^, ^8^, ^9^, ^10^, ^11^, ^12^, ^13^, ^14^, ^15^ However, even if patients initially respond well to ALK‐TKI, the majority eventually experience disease progression due to various mechanisms, including secondary mutations within the ALK tyrosine kinase domain and activation of alternative signaling pathways.^4^, ^16^, ^17^, ^18^, ^19^, ^20^, ^21^, ^22^, ^23^, ^24^, ^25^

Next‐generation ALK‐TKIs have been reported to overcome some secondary mutations mediating resistance to crizotinib.^4^, ^24^, ^25^, ^26^, ^27^ Importantly, each ALK‐TKI has different sensitivity to secondary mutations, and the clinical significance of rebiopsy to clarify the secondary mutation has been reported in various studies.^5^, ^24^, ^25^, ^26^ For instance, L1196M, which is a common gatekeeper mutation in ALK‐positive NSCLC, is resistant to crizotinib and sensitive to next generation ALK‐TKIs. On the other hand, it was reported that the solvent front ALK G1202R and compound ALK C1156Y/L1198F mutations are only sensitive to lorlatinib and crizotinib, respectively.^5^, ^24^, ^25^, ^26^

Thus, rebiopsy in ALK‐TKI‐refractory patients has attracted increasing attention as it assists in determining optimal treatment strategy; however, it is unclear whether or not rebiopsy should be performed to improve the prognosis of the ALK‐positive NSCLC patients.

The objective of this study was to analyze the significance of sequential therapy with ALK‐TKI based on the results of rebiopsy in ALK‐positive NSCLC patients.

## Methods

Patients with advanced or recurrent, histologically‐confirmed ALK‐positive NSCLC, who received ALK inhibitors in clinical practice at National Kyushu Cancer Center from March 2007 to April 2018 were included in this retrospective analysis. The ALK status of the patients was confirmed by immunohistochemistry, fluorescence in situ hybridization, or a reverse‐transcriptase polymerase chain reaction (RT‐PCR). Positivity of any of these tests indicated the rearrangement of the ALK gene. In addition, ALK fusion variants and secondary mutations were analyzed by a RT‐PCR and direct‐sequencing.^19^

We retrospectively evaluated the patient characteristics, treatment regimens, and clinical outcomes. Data on the treatment patterns and outcomes were collected from medical records.

Tumor responses were assessed with computed tomography (CT), magnetic resonance imaging (when clinically indicated), and positron emission tomography‐CT (when clinically indicated), before and during treatment, and were repeated approximately every two months. Responses were classified as progressive disease, stable disease, partial response, complete response or nonevaluable, on the basis of response evaluation criteria in solid tumors (RECIST) 1.1.

A rebiopsy was performed in the lesions that had shown progression. Based on the rebiopsy results, secondary mutations in the ALK gene that were shown to be associated with the efficacy of ALK‐TKI therapy in the preclinical or clinical setting were defined as “sensitive mutations (SM)” (i.e., alectinib to L1196M).^4^, ^24^, ^25^ The sensitivity of the secondary mutations in the 20 cases to each ALK‐TKI is shown in Table 1. In most cases, lorlatinib had sensitivity to the secondary mutations, but lorlatinib was not available at the time in some cases. Suitable and available ALK‐TKIs were selected based on the rebiopsy findings. A resistance mutation analysis was performed using direct sequencing with capillary electrophoresis of biopsy samples obtained after ALK‐TKI treatment.

**Table 1:** The sensitivity of anaplastic lymphoma kinase‐tyrosine kinase inhibitors to secondary mutations in the 20 cases

	Crizotinib	Ceritinib	Alectinib	Lorlatinib
L1196M	Resistant	Sensitive	Resistant	Sensitive
I1171T	Resistant	Sensitive	Sensitive	Sensitive
I1171N	Resistant	Sensitive	Resistant	Sensitive
G1269A	Resistant	Sensitive	Sensitive	Sensitive
G1202R	Resistant	Resistant	Resistant	Sensitive
G1123S + C1156Y	Resistant	Resistant	Resistant	Resistant
C1156Y + G1123S	Resistant	Resistant	Resistant	Resistant

This retrospective study was approved by the Ethics Committee of National Kyushu Cancer Center (2013–77), and conducted in accordance with the Declaration of Helsinki.

## Results

### Clinicopathological characteristics of patients

Seventy‐one patients who had ALK‐positive NSCLC were treated with ALK‐TKIs. Among these, 20 ALK‐positive patients underwent rebiopsy after disease progression on an ALK‐TKI. The median follow‐up period was 37.1 months. The characteristics of the patients are shown in Table 2.

**Table 2:** The clinical characteristics of the patients treated with ALK‐TKIs

		Total	Secondary sensitive mutation* (+)	Secondary sensitive mutation* (−)
		(*n* = 20)	(*n* = 8)	(*n* = 12)
Median age (years)	Median	45	42.5	50
	Range	27–68	27–53	40–67
Sex	Male	8 (40.0%)	2 (25.0%)	6 (50.0%)
	Female	12 (60.0%)	6 (75.0%)	6 (50.0%)
ECOG PS	0	12 (60.0%)	4 (50.0%)	8 (66.7%)
	1	7 (35.0%)	4 (50.0%)	3 (25.0%)
	2	1 (5.0%)	0 (0.0%)	1 (8.3%)
Smoking history	Never	16 (80.0%)	8 (100.0%)	8 (66.7%)
	Former/current	4 (20.0%)	0 (0.0%)	4 (33.3%)
Histology	Adenocarcinoma	20 (100.0%)	8 (100.0%)	12 (100.0%)
Clinical stage	III	1 (5.0%)	1 (12.5%)	0 (0.0%)
	IV	18 (90.0%)	6 (75.0%)	12 (100.0%)
	Postoperative recurrence	1 (5.0%)	1 (12.5%)	0 (0.0%)
ALK variants	Variant 1	7 (35.0%)	2 (25.0%)	5 (41.7%)
	Variant 2	6 (30.0%)	4 (50.0%)	2 (16.7%)
	Variant 3	4 (20.0%)	1 (12.5%)	3 (25.0%)
	Variant 5	2 (10.0%)	0 (0.0%)	2 (8.3%)
	Variant 1 + 6	1 (5.0%)	1 (12.5%)	0 (0.0%)

ALK, anaplastic lymphoma kinase; ECOG PS, Eastern Cooperative Oncology Group performance status; TKI, tyrosine kinase inhibitor.

The median age of the patients was 45 years (range: 28–68 years). There were eight male patients (40.0%) and 12 female patients (60.0%). Four patients (20.0%) had a history of smoking. Eighteen patients were diagnosed with clinical stage IV (90.0%), one with clinical stage III (5.0%), and one with postoperative recurrence (5.0%). The histological diagnosis was adenocarcinoma in all 20 patients. In addition, variants 1, 2, 3, and others were detected by a RT‐PCR before first line treatment in seven (35.0%), six (30.0%), four (20.0%), and three (5.0%) patients, respectively. Four (20.0%) patients underwent three or more biopsies after disease progression.

### Treatment

In first line treatment, 16 (80.0%), and four (20.0%) patients received cytotoxic chemotherapy and ALK‐TKI therapy, respectively. With regard to the ALK‐TKIs that were administered, nine (45.0%), nine (45.0%), and two (10.0%) patients received crizotinib, alectinib, and ceritinib as the first ALK‐TKI treatment.

**Figure 1:**
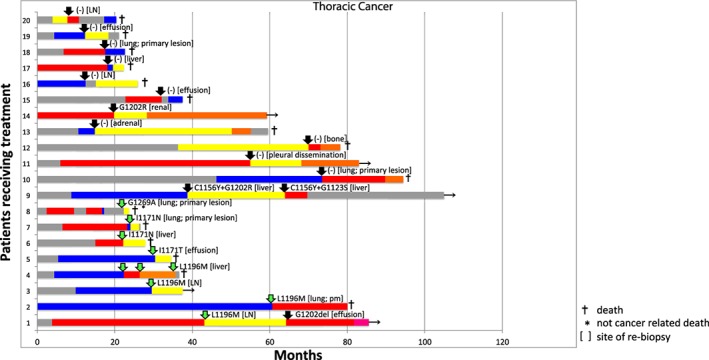
Individual swimmer plots (time to treatment failure) of 20 patients who underwent rebiopsy. In cases 1–8, the suitable anaplastic lymphoma kinase‐tyrosine kinase inhibitor (ALK‐TKI) sensitive to secondary mutation was selected based on the results of rebiopsy. In cases 9–20, no secondary mutation sensitive to ALK‐TKI was detected, and chemotherapy or remaining ALK‐TKI was selected. (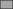) Chemotherapy, (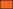) Alectinib, (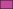) Compound B, (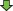) secondary sensitive mutation (+), (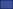) Crizotinib, (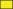) Ceritinib, (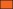) Lorlatinib, and (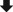) secondary sensitive mutation (‐).

Regarding the number of ALK‐TKIs administered, eight (40.0%) and 12 (60.0%) patients received two and three ALK‐TKIs, respectively. Fifteen (75.0%) received crizotinib, 15 (75.0%) received alectinib, and 15 (75.0%) received ceritinib, six (30.0%) received lorlatinib. Detailed information regarding the treatment patterns is shown in Figure 1 with individual swimmer plots (time to treatment failure) for all patients.

### Molecular characteristics of patients undergoing rebiopsy

The results of rebiopsy were used for the evaluation of the mechanisms of ALK‐TKI resistance. In the rebiopsied specimen, the original *ALK* rearrangement existed in all 20 patients. Secondary mutations were identified in 10 of all 24 biopsy specimens (41.7%). Secondary mutations included I1171N (*n* = 2), I1171T (*n* = 1), G1296A (*n* = 1), L1196M (*n* = 5), G1202deletion (*n* = 1), G1123S + C1156Y and C1156Y + G1202R (*n =* 1 [in the same one case at the second and third biopsy, respectively]). The individual responses to the next ALK‐TKI of each patient who received a repeat biopsy are listed in Tables 3 and 4 and Figure 1.

**Table 3:** Detailed information on each patient who underwent a rebiopsy (patients with a sensitive mutation at the first rebiopsy)

Patient No.	ALK‐TKI (s) prior to rebiopsy	Pattern of progression on the immediate preceding ALK‐TKI	Rebiopsy result (biopsy site) SM/not SM to next‐line ALK‐TKI	Immediate next‐line therapy	Treatment‐related adverse events (Grade 3 or 4)	Response by RECIST 1.1	PFS (months)	Pattern of progression (including the rebiopsy site or not)
1–1	Alectinib	Extracranial	L1196M (LN) SM	Ceritinib (750 → 300 mg/day)	Diarrhea (Grade 3) AST increased (Grade 3)	PR	21.0	Extracranial (not including)
1–2	Ceritinib	Extracranial	G1202del (effusion) not SM	Alectinib (600 mg/day)	‐	PR	7.5	Extracranial (including)
2	Crizotinib	Extracranial	L1196M (PM) SM	Alectinib (600 mg/day)	‐	PR	19.4	Extracranial (including)
3	Crizotinib	Extracranial	L1196M (LN) SM	Ceritinib (300 mg/day)	‐	PR	8.0	Extracranial (including)
4–1	Crizotinib	Extracranial	1196M (liver) SM	Alectinib (600 mg/day)	‐	SD	4.0	Extracranial (including)
4–2	Alectinib	Extracranial	1196M (liver) SM	Lorlatinib (100 mg/day)	‐	PR	9.3	Extracranial (including)
4–3	Lorlatinib	Extracranial	1196M (liver) not SM	Chemotherapy	‐	PD	0.9	Extracranial (including)
5	Crizotinib	Extracranial	I1171T (effusion) SM	Ceritinib (450 mg/day)	Platelet count decreased (Grade 3)	PR	4.1	CNS + extracranial (including)
6	Alectinib	Extracranial	I1171N (liver) SM	Ceritinib (300 mg/day)	‐	PR	5.6	Extracranial (not including)
7	Alectinib	CNS + extracranial	I1171N (lung) SM	Ceritinib (750 → 600 mg/day)	‐	PR	2.2	CNS + extracranial (including)
8	Aectinib, Crizotinib	Extracranial	G1269A (lung) SM	Ceritinib (750 mg)	‐	PR	1.0	Non‐cancer‐related death

ALK, anaplastic lymphoma kinase; CNS, central nervous system; LN, lymph node; PD, progressive disease; PFS, progression‐free survival; PM, pulmonary metastasis; PR, partial response; SD, stable disease; SM, sensitive mutation; RECIST 1.1, response evaluation criteria in solid tumors 1.1; TKI, tyrosine kinase inhibitor.

Ceritinib was administered between meals.

**Table 4:** Detailed information on each patient who underwent rebiopsy (patients without a sensitive mutation at the first rebiopsy)

Patient No.	ALK‐TKI (s) prior to rebiopsy	Pattern of progression on the immediate preceding ALK‐TKI	Rebiopsy result (biopsy site) SM/not SM to next‐line ALK‐TKI	Immediate next‐line therapy	Treatment‐related adverse events (Grade 3 or 4)	Response by RECIST 1.1	PFS (months)	Pattern of progression (including the rebiopsy site or not)
9–1	Crizotinib	Extracranial	C1156Y + G1202R (liver) non‐SM	Ceritinib (750 → 600 mg/day)	ALT increased (Grade 3)	SD	25.2	Extracranial (including)
9–2	Ceritinib	Extracranial	C1156Y + G1123S (liver) non‐SM	Alectinib (600 mg/day)	‐	PR	5.8	Extracranial (including)
10	Crizotinib	CNS + extracranial	No mutation (lung) non‐SM	Alectinib (600 mg/day)	‐	SD	16.2	CNS (not including)
11	Alectinib	Extracranial	G1202R (pleural dissemination) non‐SM	Ceritinib (750 → 300 mg/day)	AST increased (Grade 3) Fatigue (Grade 3)	SD	13.1	Extracranial (including)
12	Ceritinib	Extracranial	No mutation (bone) non‐SM	Alectinib (600 mg/day)	‐	SD	3.1	Extracranial (including)
13	Crizotinib	Extracranial	No mutation (LN) non‐SM	Ceritinib (600 mg/day)	‐	PR	35.0	Extracranial (including)
14	Alectinib	CNS + extracranial	No mutation (renal) non‐SM	Ceritinib (600 mg/day)	‐	SD	8.5	Extracranial (including)
15	Alectinib	Extracranial	No mutation (lung) non‐SM	Chemotherapy	‐	SD	1.7	CNS + Extracranial (including)
16	Crizotinib	CNS + extracranial†	No mutation (LN) non‐SM	Chemotherapy	‐	SD	2.7	CNS + Extracranial (including)
17	Alectinib	Extracranial	No mutation (lung) non‐SM	Crizotinib (500 mg/day)	‐	PD	1.4	Extracranial (including)
18	Alectinib	CNS + extracranial†	No mutation (lung) non‐SM	Crizotinib (500 mg/day)	‐	PD	5.1	Extracranial (including)
19	Crizotinib	CNS + extracranial†	No mutation (effusion) non‐SM	Ceritinib (450 mg/day)	‐	SD	2.1	Extracranial (including)
20	Ceritinib	Extracranial	No mutation (effusion) non‐SM	Alectinib (600 mg/day)	‐	SD	3.0	Extracranial (including)

† Stereotactic radiotherapy was performed for new brain metastases, and crizotinib or alectinib was continued beyond PD.

ALK, anaplastic lymphoma kinase; TKI, tyrosine kinase inhibitor; SM, sensitive mutation; RECIST 1.1, response evaluation criteria in solid tumors 1.1; PR, partial response; SD, stable disease; PD, progressive disease; PFS, progression free survival; CNS, central nervous system; LN, lymph node.

Ceritinib was administered between meals.

We first compared the objective responses to ALK‐TKI therapy of 20 rebiopsied patients with and without secondary SM. The objective response rate (ORR) of the cases with secondary SM who received ALK‐TKI therapy was 88.9%, while the ORR of the patients without secondary SM who received ALK TKI was 20.0% (Table 5).

**Table 5:** The results of patients who underwent repeat biopsy after ALK‐TKI failure

	Secondary sensitive mutation‡ (+) (*n* = 9)	Secondary sensitive mutation‡ (−) (*n* = 15)
ORR of the treatment after rebiopsy	88.9%	20.0%
PFS of the treatment after rebiopsy	5.6 months	5.1 months
Overall survival†	37.0 months	49.0 months

ALK, anaplastic lymphoma kinase; ORR, objective response rate; PFS, progression‐free survival; TKI, tyrosine kinase inhibitor.

† Overall survival (OS) was the time from the start of first‐line treatment until death from any cause.

‡ Secondary sensitive mutations were shown to be effective in preclinical or clinical setting on the ALK‐TKI which were used after the rebiopsy.

We next compared the treatment outcomes to sequential therapy among patients with and without secondary SM. The median progression free survival (PFS) achieved by the eight patients with nine secondary SM cases who received ALK‐TKI therapy was 5.6 months, while the median PFS of the 12 patients with 16 cases with nonsecondary SM who received next line treatment (nontailored ALK‐TKI or chemotherapy) was 5.1 months (Table 5). With regard to overall survival (OS), among the eight patients with at least one secondary SM on rebiopsy, the median OS was 37.0 months, while the median OS among the patients without any secondary sensitive ALK mutations was 49.0 months (Table 5).

Out of the 20 cases, five cases showed progression in the central nervous system (CNS) during the next line therapy. Of those, just one case (Patient ID Number 10 in Table 3, in Fig 1) showed progression in the CNS only, and the other four cases (Patient ID Numbers 5, 7, 15, 16 in Table 3, in Fig 1) showed progression in both the CNS as well as extracranial lesions.

Detailed individual data on the secondary mutations are provided in Tables 3 and 4 and Figure 1.

## Discussion

Previous studies have reported that rebiopsy could provide further information, including histological or genetic changes that might be helpful in optimizing the next treatment^24^, ^25^; however, little clinical data exists regarding the prognostic impact of rebiopsy on ALK‐positive NSCLC patients. In this retrospective analysis, we evaluated the treatment course and clinical efficacy of ALK‐TKI in ALK‐positive NSCLC patients who received rebiopsy after relapse on ALK‐TKI, and the administration of ALK‐TKIs based on the secondary sensitive mutations was associated with a high ORR and relatively short PFS (87.5% and 5.4 months, respectively).

Some clinical trials have demonstrated that there is good efficacy of second generation ALK‐TKI in comparison to chemotherapy for crizotinib‐pretreated ALK‐positive NSCLC patients.^9^, ^14^ In addition, some studies showed the remarkable efficacy of next generation ALK‐TKI tailored to the secondary mutation.^12^, ^25^, ^28^ In the current study, each patient's in vitro ALK‐TKI‐sensitivity profile and ALK resistance mutations were used to select the next ALK‐TKI for the treatment of ALK‐TKI therapy refractory patients. For example, L1196M (shown in cases 1–4, in Fig 1) and I1171N (shown in cases 6 and 7 in Fig 1) are reported to be associated with sensitivity to ceritinib, brigatinib, and lorlatinib, and resistance to crizotinib and alectinib.^24^, ^25^ Similarly, I1171T (shown in Fig 1 [case 5]) and G1296A (shown in Fig 1 [case 8]) are reported to be associated with sensitivity to second and third generation ALK‐TKIs and resistance to crizotinib.^24^, ^25^ We respectively selected the suitable ALK‐TKI based on these data, and good responses were observed in those cases (ORR: 88.9%; Fig 1 [cases 1–8] and Table 3). On the other hand, in the cases without secondary SM (Fig 1 [cases 9–20]), chemotherapy or remaining ALK‐TKI was selected, and the ORR was relatively low (20.0%); however, the PFS of the patients with SMs was relatively short (with SM vs. without SM: 5.6 months vs. 5.1 months). These results suggest that once resistance to an ALK‐TKI emerges, even when it is a secondary SM, some ALK‐independent resistance mechanisms, such as bypass signaling, might emerge in a combinatory manner or over a short period after ALK‐TKI failure, which should be clarified by comprehensive testing modalities, such as next generation sequencing. If a new treatment strategy (i.e., combination treatment with a next generation ALK‐TKI and agents targeting the bypass track) that overcomes this resistance mechanism is established, then the prognosis of ALK‐TKI refractory patients will be remarkably improved in comparison to the existing treatment. Recently, the phase II study of lorlatinib has been reported.^11^, ^29^ The cohort with at least one secondary mutation in the baseline tumor biopsy had a PFS of 11.0 months, while the cohort with no secondary mutation in the baseline biopsy had a PFS of 5.4 months.^29^ This result suggests that the PFS in the current study was relatively short; however, the previous cohorts included patients treated with not only second‐generation ALK‐TKIs, but also crizotinib as pretreatment of lorlatinib. Whether or not only crizotinib or noncrizotinib ALK‐TKIs were administered as pretreatment might affect the efficacy of lorlatinib. Among the patients treated only with crizotinib as pretreatment of lorlatinib, the median PFS of lorlatinib was not reached (95% confidence interval [CI]: 12.5 months–not reached), while among those treated with noncrizotinib ALK‐TKIs as pretreatment of lorlatinib, the median PFS of lorlatinib was 5.5 months (95% C.I., 2·7–9·0 months).^11^ In the current study, nine cases were treated only with crizotinib before rebiopsy, while 11 cases were treated with ALK‐TKIs including noncrizotinib ALK‐TKIs before the rebiopsy. This might be the one reason for the short PFS in the current study. In addition, in the phase II study, lorlatinib was administered in all cases, whereas in the current study, the most frequently administered tailored ALK‐TKI was ceritinib, and most cases with pretreatment of ALK‐TKIs received alectinib. This might also explain the relatively short PFS in the current study.

Regarding CNS disease, five of the 20 cases after rebiopsy showed progression in the CNS during next‐line therapy. Of those, one case showed progression in the CNS only, and the other four showed progression in both the CNS as well as extracranial regions. Therefore, progression in the CNS might be a source of bias that affected the PFS in the current study. We propose to perform detailed analyses to clarify the efficacy of tailored ALK‐TKIs against CNS lesions that progress on alectinib in a larger population.

Next generation ALK TKIs, such as alectinib and brigatinib, showed superior efficacy in the primary treatment of ALK‐positive NSCLC compared with crizotinib.^10^, ^11^ However, as shown in Figure 1, the PFS of patients who received next‐generation ALK‐TKIs tailored to secondary mutations is relatively short, and the PFS of patients receiving next‐generation ALK‐TKIs in first‐line treatment is expected to be long. Patients with alectinib‐refractory ALK‐positive NSCLC who received brigatinib also showed relatively short PFS, even though the cases had an SM to brigatinib.^12^ Using better and new‐generation ALK‐TKIs on a priority basis might improve the prognosis of patients with ALK‐positive NSCLC. Yoda *et al*. also mentioned that the emergence of secondary resistance mutations could be prevented with upfront treatment using the third‐generation inhibitor lorlatinib.^25^ The results of an ongoing randomized phase 3 trial comparing lorlatinib to crizotinib as first‐line therapy for advanced ALK‐positive lung cancer (NCT03052608) might partly support this hypothesis.

The present study had several noteworthy limitations. It was a retrospective analysis performed in a single institution. The retrospective nature of the study might have induced a selection bias, and the duration of follow‐up was also limited. Furthermore, the number of patients who received repeated biopsy was small. Thus, additional prospective analyses of ALK‐positive patients are needed to confirm these findings. In addition, resistance mechanisms are sometimes heterogeneous between lesions in a single patient, and the resistance mechanisms were analyzed in the rebiopsy lesions only; however, it is not realistic to perform a rebiopsy for all lesions. With regard to the difference in the OS between patients with and without SMs, the year of the diagnosis may have influenced survival because of differences in the ALK‐TKIs that were available at that time; however, ALK‐TKIs sensitive to the secondary mutation based on the rebiopsy results were selected, and the efficacy of ALK‐TKIs tailored to secondary mutations targeted for analysis in the current study.

## Conclusion

The selection of ALK‐TKI based on secondary SM was associated with a high ORR and relatively short PFS. Using better and new generation ALK‐TKIs on a priority basis could improve the prognosis of ALK‐positive NSCLC patients, and the mechanism responsible for the short PFS of sensitive ALK‐TKI to secondary mutation should be clarified. Larger‐scale and well‐controlled prospective studies should be performed to confirm these observations.

## Disclosure

All authors had full access to the data in the study and take responsibility for its integrity and the accuracy of the data analysis.

Dr Seto reports grants from Bayer Yakuhin, grants from Eisai, grants from Merck Serono, grants from Novartis Pharma, personal fees from Bristol‐Myers Squibb, personal fees from Kyowa Hakko Kirin, personal fees from Mochida Pharmaceutical, personal fees from Ono Pharmaceutical, personal fees from Roche Singapore, personal fees from Showa Yakuhin, personal fees from Taiho Pharmaceutical, personal fees from Takeda Pharmaceutical, grants and personal fees from Astellas Pharma, grants and personal fees from AstraZeneca, grants and personal fees from Chugai Pharmaceutical, grants and personal fees from Daiichi Sankyo, grants and personal fees from Eli Lilly Japan, grants and personal fees from Kissei Pharmaceutical, grants and personal fees from MSD, grants and personal fees from Nippon Boehringer Ingelheim, grants and personal fees from Pfizer Japan, grants and personal fees from Yakult Honsha, outside the submitted work. D. Nosaki reports grants from Novartis Pharma, personal fees from AstraZeneca, personal fees from Chugai Pharmaceutical, personal fees from Eli Lilly Japan, personal fees from Kyowa Hakko Kirin, personal fees from Nippon Boehringer Ingelheim, personal fees from Nippon Kayaku, personal fees from Ono Pharmaceutical, personal fees from Pfizer Japan, personal fees from Taiho Pharmaceutical, grants and personal fees from MSD, outside the submitted work. Dr. Takenoyama reports grants and personal fees from AstraZeneca, grants and personal fees from Bristol‐Myers Squibb, grants and personal fees from Chugai Pharmaceutical, grants and personal fees from Eli Lilly Japan, grants and personal fees from Nippon Boehringer Ingelheim, grants and personal fees from Ono Pharmaceutical, grants and personal fees from Taiho Pharmaceutical, personal fees from MSD, grants from Johnson & Johnson, grants from Kaketsuken, grants from Novartis Pharma, grants from Yakult Honsha, outside the submitted work.

All other authors declare no competing interests.

## References

[1] Shaw AT , Kim DW , Nakagawa K *et al* Crizotinib versus chemotherapy in advanced ALK‐positive lung cancer. N Engl J Med 2013; 368: 2385–94. 23724913 10.1056/NEJMoa1214886

[2] Seto T , Kiura K , Nishio M *et al* CH5424802 (RO5424802) for patients with ALK‐rearranged advanced non‐small‐cell lung cancer (AF‐001JP study): A single‐arm, open‐label, phase 1‐2 study. Lancet Oncol 2013; 14: 590–8. 23639470 10.1016/S1470-2045(13)70142-6

[3] Asao T , Fujiwara Y , Itahashi K *et al* Sequential use of anaplastic lymphoma kinase inhibitors in Japanese patients with ALK‐rearranged non‐small‐cell lung Cancer: A retrospective analysis. Clin Lung Cancer 2017; 18: e251#x2013;8. 28065466 10.1016/j.cllc.2016.11.015

[4] Friboulet L , Li N , Katayama R *et al* The ALK inhibitor Ceritinib overcomes Crizotinib resistance in non‐small cell lung Cancer. Cancer Discov 2014; 4: 662–73. 24675041 10.1158/2159-8290.CD-13-0846 PMC4068971

[5] Zhang S , Anjum R , Squillace R *et al* The potent ALK inhibitor Brigatinib (AP26113) overcomes mechanisms of resistance to first‐ and second‐generation ALK inhibitors in preclinical models. Clin Cancer Res 2016; 22: 5527–38. 27780853 10.1158/1078-0432.CCR-16-0569

[6] Hida T , Nokihara H , Kondo M *et al* Alectinib versus crizotinib in patients with ALK‐positive non‐small‐cell lung cancer (J‐ALEX): An open‐label, randomised phase 3 trial. Lancet 2017; 390: 29–39. 28501140 10.1016/S0140-6736(17)30565-2

[7] Kim DW , Tiseo M , Ahn MJ *et al* Brigatinib in patients with Crizotinib‐refractory anaplastic lymphoma kinase‐positive non‐small‐cell lung Cancer: A randomized, Multicenter phase II trial. J Clin Oncol 2017; 35: 2490–8. 28475456 10.1200/JCO.2016.71.5904

[8] Ito K , Hataji O , Kobayashi H *et al* Sequential therapy with Crizotinib and Alectinib in ALK‐rearranged non‐small cell lung Cancer ‐ A Multicenter retrospective study. J Thorac Oncol 2017; 12: 390–6. 27498387 10.1016/j.jtho.2016.07.022

[9] Shaw AT , Kim TM , Crino L *et al* Ceritinib versus chemotherapy in patients with ALK‐rearranged non‐small‐cell lung cancer previously given chemotherapy and crizotinib (ASCEND‐5): A randomised, controlled, open‐label, phase 3 trial. Lancet Oncol 2017; 18: 874–86. 28602779 10.1016/S1470-2045(17)30339-X

[10] Peters S , Camidge DR , Shaw AT *et al* Alectinib versus Crizotinib in untreated ALK‐positive non‐small‐cell lung Cancer. N Engl J Med 2017; 377: 829–38. 28586279 10.1056/NEJMoa1704795

[11] Solomon BJ , Besse B , Bauer TM *et al* Lorlatinib in patients with ALK‐positive non‐small‐cell lung cancer: Results from a global phase 2 study. Lancet Oncol 2018; 19: 1654–67. 30413378 10.1016/S1470-2045(18)30649-1

[12] Lin JJ , Zhu VW , Schoenfeld AJ *et al* Brigatinib in patients with Alectinib‐refractory ALK‐positive NSCLC. J Thorac Oncol 2018; 13: 1530–8. 29935304 10.1016/j.jtho.2018.06.005 PMC6341982

[13] Gettinger SN , Bazhenova LA , Langer CJ *et al* Activity and safety of brigatinib in ALK‐rearranged non‐small‐cell lung cancer and other malignancies: A single‐arm, open‐label, phase 1/2 trial. Lancet Oncol 2016; 17: 1683–96. 27836716 10.1016/S1470-2045(16)30392-8

[14] Novello S , Mazieres J , Oh IJ *et al* Alectinib versus chemotherapy in crizotinib‐pretreated anaplastic lymphoma kinase (ALK)‐positive non‐small‐cell lung cancer: Results from the phase III ALUR study. Ann Oncol 2018; 29: 1409–16. 29668860 10.1093/annonc/mdy121 PMC6005013

[15] Shaw AT , Felip E , Bauer TM *et al* Lorlatinib in non‐small‐cell lung cancer with *ALK* or *ROS1* rearrangement: An international, multicentre, open‐label, single‐arm first‐in‐man phase 1 trial. Lancet Oncol 2017; 18: 1590–9. 29074098 10.1016/S1470-2045(17)30680-0 PMC5777233

[16] Katayama R , Shaw AT , Khan TM *et al* Mechanisms of acquired crizotinib resistance in ALK‐rearranged lung cancers. Sci Transl Med 2012; 4: 120ra117. 10.1126/scitranslmed.3003316 PMC3385512 22277784

[17] Toyokawa G , Hirai F , Inamasu E *et al* Secondary mutations at I1171 in the ALK gene confer resistance to both Crizotinib and Alectinib. J Thorac Oncol 2014; 9: e86#x2013;7. 25393798 10.1097/JTO.0000000000000358

[18] Crystal AS , Shaw AT , Sequist LV *et al* Patient‐derived models of acquired resistance can identify effective drug combinations for cancer. Science 2014; 346: 1480–6. 25394791 10.1126/science.1254721 PMC4388482

[19] Katayama R , Friboulet L , Koike S *et al* Two novel ALK mutations mediate acquired resistance to the next‐generation ALK inhibitor alectinib. Clin Cancer Res 2014; 20: 5686–96. 25228534 10.1158/1078-0432.CCR-14-1511 PMC4233168

[20] Toyokawa G , Inamasu E , Shimamatsu S *et al* Identification of a novel ALK G1123S mutation in a patient with ALK‐rearranged non‐small‐cell lung Cancer exhibiting resistance to Ceritinib. J Thorac Oncol 2015; 10: e55#x2013;7. 26134233 10.1097/JTO.0000000000000509

[21] Toyokawa G , Seto T . Updated evidence on the mechanisms of resistance to ALK inhibitors and strategies to overcome such resistance: Clinical and preclinical data. Oncol Res Treat 2015; 38: 291–8. 26045026 10.1159/000430852

[22] Toyokawa G , Seto T , Takenoyama M , Ichinose Y . W'ALK' into the next stage. Clin Lung Cancer 2017; 18: 122–6. 27865624 10.1016/j.cllc.2016.10.005

[23] Akamine T , Toyokawa G , Tagawa T , Seto T . Spotlight on lorlatinib and its potential in the treatment of NSCLC: The evidence to date. Onco Targets Ther 2018; 11: 5093–101. 30174447 10.2147/OTT.S165511 PMC6110295

[24] Gainor JF , Dardaei L , Yoda S *et al* Molecular mechanisms of resistance to first‐ and second‐generation ALK inhibitors in ALK‐rearranged lung Cancer. Cancer Discov 2016; 6: 1118–33. 27432227 10.1158/2159-8290.CD-16-0596 PMC5050111

[25] Yoda S , Lin JJ , Lawrence MS *et al* Sequential ALK inhibitors can select for Lorlatinib‐resistant compound ALK mutations in ALK‐positive lung Cancer. Cancer Discov 2018; 8: 714–29. 29650534 10.1158/2159-8290.CD-17-1256 PMC5984716

[26] Shaw AT , Friboulet L , Leshchiner I *et al* Resensitization to Crizotinib by the Lorlatinib ALK resistance mutation L1198F. N Engl J Med 2016; 374: 54–61. 26698910 10.1056/NEJMoa1508887 PMC4773904

[27] Lin JJ , Riely GJ , Shaw AT . Targeting ALK: Precision medicine takes on drug resistance. Cancer Discov 2017; 7: 137–55. 28122866 10.1158/2159-8290.CD-16-1123 PMC5296241

[28] Lin JJ , Zhu VW , Yoda S *et al* Impact of EML4‐ALK variant on resistance mechanisms and clinical outcomes in ALK‐positive lung Cancer. J Clin Oncol 2018; 36: 1199–206. 29373100 10.1200/JCO.2017.76.2294 PMC5903999

[29] Shaw AT , Solomon BJ , Besse B *et al* ALK resistance mutations and efficacy of Lorlatinib in advanced anaplastic lymphoma kinase‐positive non‐small‐cell lung cancer. J Clin Oncol 2019; 37: 1370–1379. 30892989 10.1200/JCO.18.02236 PMC6544460

